# Photobactericides—A Local Option against Multi-Drug Resistant Bacteria

**DOI:** 10.3390/antibiotics2020182

**Published:** 2013-03-27

**Authors:** Mark Wainwright, Leonard Amaral

**Affiliations:** 1 School of Pharmacy & Biomolecular Sciences, Liverpool John Moores University, Liverpool L3 3AF, UK; 2 Travel Medicine of the CMDT, Institute of Hygiene & Tropical Medicine, Universidade Nova de Lisboa, 100 Rua Junqueira, 1349-008 Lisbon, Portugal; E-Mail: lamaral@ihmt.unl.pt

**Keywords:** photobactericide, conventional resistance mechanisms, methylene blue

## Abstract

The use of light-activated bactericidal agents—photobactericides—is suggested in local infection in order to conserve conventional antibacterials for more systemic disease. Local administration of a photobactericide such as methylene blue coupled with locally-targeted red light illumination ensures the production of non-specific reactive oxygen species and thus a rapid and localised antibacterial response, regardless of the conventional resistance status. To this end, the response of photobactericides to conventional resistance mechanisms, and their potential use in infection, is discussed.

## 1. Introduction

Given the bewildering numbers of drug-resistant strains of bacteria in modern healthcare, it is clear that conventional antibacterial approaches are no longer widely effective. This is truly a terrible situation when considered from the viewpoint of those involved in producing the “Golden Age” of antibiotics when all of mankind’s infectious diseases were thought to be susceptible to Fleming’s legacy. However, in the early 21st Century, with the benefit of considerable hindsight, there is a sound understanding of the failure of conventional antibacterial chemotherapy, based with greater emphasis on bacterial evolution than on the initial good fortune and subsequent—if predictable—egotism of *Homo sapiens*. This understanding counts the rapidity of bacterial genetic turnover and adaptability as key, thus explaining the apparent ease—on a human timescale, at least—with which our bacterial colonists become immune to the chemical battery deployed against them.

It is not in the least surprising that the early, successful antibacterial types such as the sulphonamides and penicillins were used with such alacrity. In the period 1935–1945 patient mortality from bacterial disease was high, and obviously there was a massive requirement for wound and infection therapy during World War II. However, the phenomenon of drug resistance was appreciated at least by those involved in the field of chemotherapy—Fleming himself alluded to it in his Nobel Prize Lecture in 1945 [1]. Despite this, antibacterial drugs have been used, even in well-organised healthcare systems, with breathtaking profligacy until relatively recently. Again, with hindsight, bacterial evolutionary kinetics have *always* meant that specific antibacterial drugs, or drug classes, would have a finite period of usefulness.

Despite this deterministic outlook, our clinical use of antibacterial agents has been flawed. Investigation of the modes of action of these valuable commodities has, in most cases, shown that each drug type has a single site and mode of action—for example, the mimicking of the D-alanine-D-alanine terminus of the incipient peptide crosslink in bacterial cell wall peptidoglycan by the bicyclic penicillin nucleus, which leads to the inhibition of action of bacterial transpeptidase. Resistance to the initially-introduced penicillin drugs was seen in the immediate post-war period due to endogenous hydrolytic enzymes, later known as β-lactamases, the overexpression of which furnished mutant organisms with an evolutionary advantage, and the adaptation of which allowed the clinical survival of their progeny. While early penicillin resistance was particularly problematic in Gram-positive cocci [2], one of the most significant clinical threats in recent years can be thought of as an elaboration in Gram-negative pathogens, *i.e.*, the extended spectrum β-lactamases (ESBLs) which inactivate the carbapenems, important modern drugs expensively developed for use against serious Gram-negative infection [3]. These again obey the single site/single mode of action paradigm, and act in the same way as the original penicillins developed by Florey and the Oxford group in the 1940s.

A further problem in drug resistance lies in the ability of bacteria to remove or to exclude toxic substances. Generally cells are able to expel xenobiotic substances from the interior using protein pumps, and in drug resistant forms, this facility can be over expressed. However, given the relatively non-specific nature of the substrates involved this may be considered to be a far greater problem in terms of drug therapy since the potential structural range of candidate therapeutics is wide, *i.e.*, efflux capability does not pertain to a single chemical class of agent. Similarly, the exclusion—*i.e.*, non-admittance-of antibacterial agents from the cell is normally achieved via the lowered expression of small porin channels in the cell exterior. Again, this can have no relevance to a particular chemical class of therapeutic.

Thus, 21st Century bacterial infection often represents a considerable—and increasingly insurmountable—problem to those administering the conventional armamentarium. In addition, new drugs are very slow in arriving, and most which have been accepted for clinical use still obey the single site/single mode of action paradigm. Consequently, not only will these drugs have a limited period of utility, they will also add to the selective evolutionary pressure already driving bacterial resistance development.

The photoantimicrobial approach to infectious disease offers multifactorial attack—*i.e.*, multiple and variable sites of action coupled with a non-selective, oxidative mode of action. As the term suggests, photoantimicrobials require light activation so local application and activation are required, but this has important ramifications regarding treatment, particularly from the point of view of effects on local flora in comparison to those seen with conventional systemics, and in the lack of resistance development.

**Figure 1:**
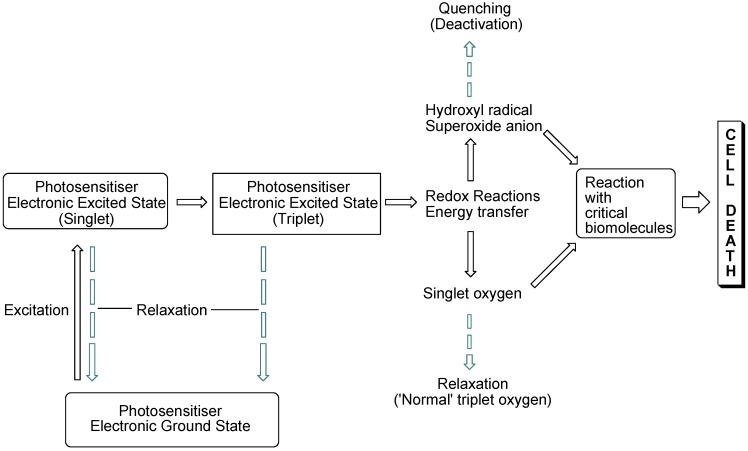
Photosensitisation pathways leading to bacterial cell death. Dashed arrows indicate possible deactivational (non-destructive) routes.

The interaction of light with photosensitising molecules, of which the photoantimicrobials are a sub-class, is slightly different to that of the larger group of dyes and pigments. Light absorption by both classes of compounds entails the removal of a certain wavelength range of the incident light energy, depending on the chemical make-up of the absorber, leading to electronic excitation. In dyes and pigments, the excitational energy is lost rapidly, whereas in photosensitiser molecules the excited state is sufficiently long-lived for electronic rearrangement to occur, promoting electron transfer reactions and the transfer of excitational energy to molecular oxygen. Both of these routes result in the formation of reactive oxygen species which are highly damaging in the cellular milieu. However, they are short-lived species, so oxidative damage is highly localised (Figure 1).

## 2. Photoantimicrobial Chemistry

**Figure 2:**
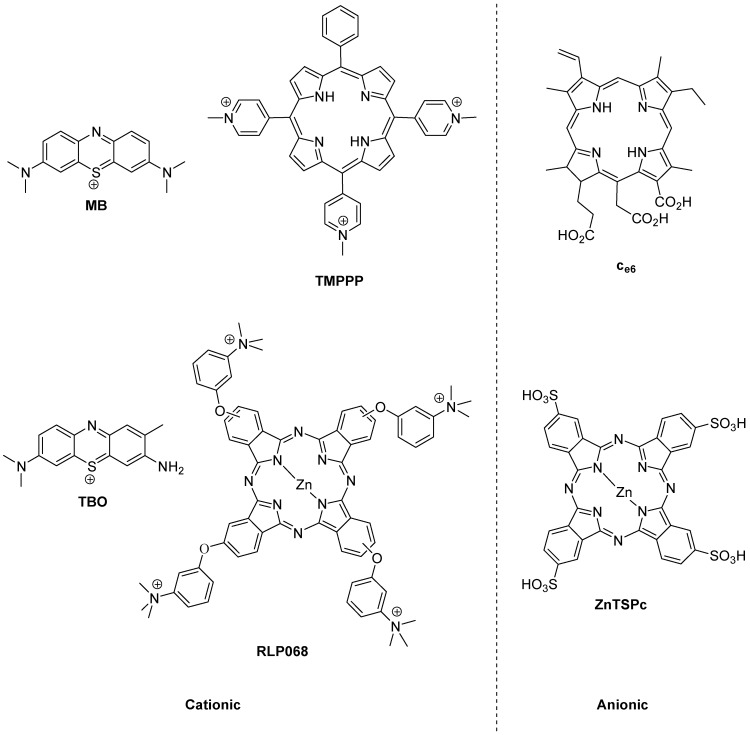
Photobactericides from the main classes of photosensitiser. Methylene blue (MB) and toluidine blue (TBO), phenothiazinium class; TMPPP [tri(*N*-methylpyrid-4-yl)phenylporphyrin] and C_e6_, porphyrin; RLP068 and ZnTSPc, phthalocyanine. Cationic examples are broad-spectrum, anionic examples are active mainly against Gram-positive bacteria.

The history of photoantimicrobials is based on the observed antimicrobial activity of a handful of dyes which were associated with the burgeoning science of biological staining at the end of the 19th Century. The lead compound resulting from this was the phenothiazinium derivative methylene blue (MB, Figure 2), which was shown to kill bacteria, viruses, fungi and protozoa under illumination during the same momentous period (1928–1935) which covered both Fleming’s and Domagk’s major antibacterial discoveries. There remains a strong rationale for the use of methylene blue as a chemical lead by researchers involved in photoantimicrobial discovery, as it also represents the first-in-clinic example, being currently licensed for oral disinfection [4].

The reason for the truly broad antibacterial spectrum of methylene blue and its congeners lies in the possession of a permanent positive charge. This guarantees the activity against Gram-negative bacteria not seen in anionic (negatively charged) or neutral photosensitisers, such as the haematoporphyrin derivatives used far more successfully in the photodynamic therapy of cancer (PDT) [5].

However, broad-spectrum photoantibacterial activity is not limited to the phenothiazinium class. Indeed it should not be limited by chemical class at all. The relevant criterion here is that the photosensitiser in question has a positive charge. Consequently, there are examples of broad-spectrum photoantibacterials in the synthetic porphyrin and phthalocyanine classes also (Figure 2) [6]. 

Another important aspect in the development of a clinically-useful photobactericide is the associated absorption spectrum. The reason for this is the presence of other absorbing species at the infection site. Such species are the natural materials present—e.g., blood in a wound contains haem pigments which absorb both ultraviolet and visible wavelengths, while soluble aminoacids and vitamins absorb only in the ultraviolet region. A considerable portion of photobactericidal research is thus entailed in the production of molecules which may be excited at longer wavelengths. Practically, since the longest wavelength of absorption of haem is a weak band at 630 nm, intense absorbers beyond this wavelength may be easily discovered among both the phenothiazinium and phthalocyanine series, e.g., methylene blue (660 nm) and RLP068 (668 nm) respectively (Figure 2). Clearly efficient excitation is also necessary, but this is relatively straightforward given access to diode lasers and light-emitting diode-based equipment.

## 3. Photosensitiser Activity against Resistant Bacteria

Logically, a structure-specific mechanism of resistance such as β-lactamase activity requires that structural motif for activity. Conventional agents not containing this motif should be immune to attack. Similarly drug activity may be nullified by the alteration of the active site, for example in tetracycline resistance via ribosomal protection. In either of these scenarios, substitution of the original therapeutic with one which is chemically distinct (*i.e.*, from a different class), should regain efficacy. However, where there is more than one resistance mechanism at work—as is increasingly the case—this approach becomes less successful. The utility of photoantimicrobial agents lies in efficacy *regardless* of the conventional drug resistance mechanism. This is considered below.

## 4. Target Alteration

As noted above, target alteration as a drug resistance mechanism usually pertains to changes to minor morphological or chemical changes which result in lowered affinity of the drug for its target, for example ribosomal protection in tetracycline resistance [7], or the change from D-alanine-D-alanine to D-alanine-D-lactate at the glycopeptide-active site in the developing bacterial cell wall in vancomycin-resistant enterococci [8]. Such changes are specific for the incoming drug molecule and may often thus confer class resistance.

The effects of a photobactericide in such a case should be consistent between drug-sensitive and drug-resistant cells. This is due to structural dissimilarity between the photobactericidal molecule and conventional drugs, and to the non-specific nature of the oxidising species produced on illumination. Such activity has been reported for methylene blue derivatives against vancomycin-susceptible and vancomycin-resistant enterococci [9].

## 5. Drug Inactivation

As with target alteration, drug inactivation as a resistance mechanism may rely on target discrimination. The β-lactamases provide an excellent example of this, being inactive against other chemical classes of antibacterial agent. Clearly the same argument pertains for photobactericidal activity in such cases, given the difference in chemical structures employed and also in the reactive oxygen species produced, and effective photobactericidal activity has been reported for methylene blue against extended-spectrum β-lactamase (ESBL)-producing strains of *Escherichia coli* [10]. Such activity may also be expected against New Delhi metallo-β-lactamase-1 producing bacteria, underlining the utility of the light-activated approach.

**Figure 3:**
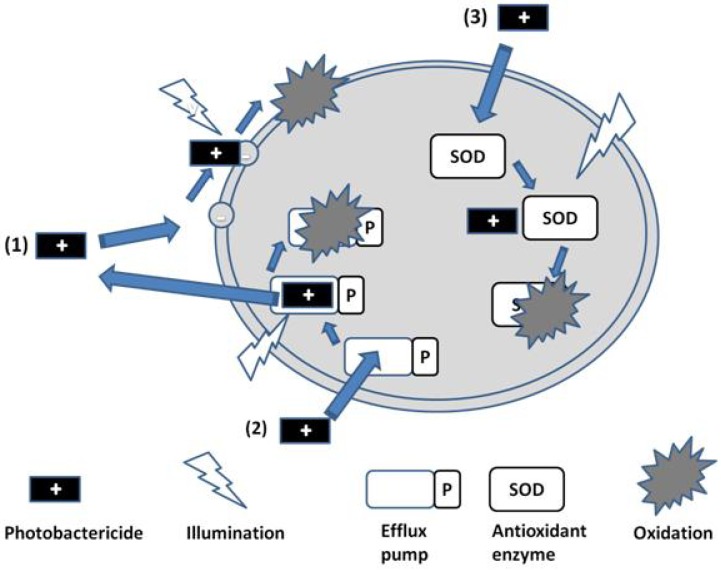
Photobactericide action and resistance mechanisms.

It might be expected that antioxidant enzymes would present a resistance route against photobactericidal agents, acting to nullify the reactive oxygen species produced on illumination. However, it has been demonstrated that enzymes such as catalases, peroxidases and superoxide dismutase are themselves inactivated on exposure to singlet oxygen (SOD, Route 3, Figure 3) [11]. It is therefore not surprising that upregulation of SOD as a consequence of *Staphylococcus aureus* exposure to protoporphyrin-initiated photodynamic treatment reportedly does not affect the cell-killing outcome [12].

## 6. Decreased Cell Permeability

In comparison to the two previous routes, decreased cell permeability is much less structure specific, being based on significantly-reduced numbers of porins in the outer membrane of Gram-negative bacteria. Consequently, most conventional antibacterial agents are effectively barred from their targets. However, it has been demonstrated for cationic photobactericides, such as the pyridinium phthalocyanines, that they gain entry to the Gram-negative cell via self-promoted uptake [13]. This process involves the disruption of the outer membrane by the positive charge, or charges, on the photosensitiser via displacement of the divalent metal ions required for the stabilisation of the membrane’s anionic head groups. Subsequently, the oxidising species produced by photobactericides may then produce non-specific oxidation, causing damage to the outer membrane sufficient to cause catastrophic breakdown to a similar extent to that caused by peptide antibiotics such as the polymyxins. Significant disturbance to the outer membrane will also allow ingress to the interior of the cell and photodamage to underlying structures/organelles [14]. While anionic photosensitisers are generally ineffective against Gram-negative bacteria, this may be reversed by attaching a polycationic residue to the photosensitiser. This has been reported for chlorin e6 (C_e6_, Figure 2) and polyethyleneimines [15].

In addition, it has been established for a considerable time that the exclusion of a photobactericide from the target cell does not inhibit cell damage, since polymer-bound examples have been shown to cause cell death on illumination, allowing the development of photoantimicrobial plastics and textiles [16,17].

## 7. Overexpression of Efflux Pumps

A similar argument can be made for the assisted exclusion of photobacterides from the target cell, although Hamblin has shown that both the phenothiazinium derivatives methylene blue and toluidine blue (MB and TBO, respectively, Figure 2) can act as substrates for efflux pumps in bacteria [18], and that this behaviour may be reversed via the addition of inhibitors such as verapamil [19]. Other cationic photosensitisers, from different chemical classes of significantly greater moleclular weight, such as the porphyrins and phthalocyanines are reported to avoid transport by such efflux pumps. In addition, it is theoretically possible to inactivate the efflux pump via illumination during transport of the photosensitiser, as indicated in Figure 3 (Route 2). This is an approach currently under investigation.

It is emphasised that, unlike conventional antibacterial agents, photobactericides are intended for local/topical application—*i.e.*, where there is a focus of infection rather than dissemination. Clearly local application allows for concentration of the active agent at the site of infection, rather than relying on transport through the bloodstream following systemic administration. Light is also applied in a focused manner. In terms of the major resistance mechanisms discussed above, only that involving exclusion via efflux appears to offer any difficulty for the photobactericidal approach, and this has not been encountered in the clinic. In addition, local administration to an infection site should produce a higher concentration of photosensitiser external to the target cell than would be required for cell death—it should be recalled that photobactericides can produce reactive oxygen species on illumination outside the cell.

The multiple site of action/mode of action paradigm associated with photoantimicrobial agents is underlined in reported passaging experiments. For example, twenty daily passages using the cationic phthalocyanine RLP068 (Figure 2) against strains of *Staphylococcus aureus* and *Pseudomonas aeruginosa,* with a range of conventional drug resistance profiles, demonstrated no development of resistance to the photodynamic approach [20].

Given the demonstrable efficacies of various photobactericides against a broad-spectrum of bacterial pathogens, regardless of conventional resistance status, the clinical potential of this class of agents is considerable. For clinicians it is, however, apparently tempered by the fact that light activation is required—this being seen as a deviation from the standard antibacterial paradigm. It should be pointed out both that the standard paradigm is no longer successful and that light activation is routinely used in other areas of medicine, such as the treatment of psoriasis with PUVA. Clearly, if mankind is to keep up with its bacterial colonists, changes will need to be made to the clinical approach to microbial—not just bacterial—disease, and photobactericides should be part of that change.
